# Hypernatremia in Dice Snakes (*Natrix tessellata*) from a Coastal Population: Implications for Osmoregulation in Marine Snake Prototypes

**DOI:** 10.1371/journal.pone.0092617

**Published:** 2014-03-21

**Authors:** François Brischoux, Yurii V. Kornilev

**Affiliations:** 1 Centre d'Etudes Biologiques de Chizé, CEBC UMR 7372 CNRS-ULR, Villiers en Bois, France; 2 Bulgarian Society for the Protection of Birds, NCC “Poda”, Burgas, Bulgaria; State Natural History Museum, Germany

## Abstract

The widespread relationship between salt excreting structures (e.g., salt glands) and marine life strongly suggests that the ability to regulate salt balance has been crucial during the transition to marine life in tetrapods. Elevated natremia (plasma sodium) recorded in several marine snakes species suggests that the development of a tolerance toward hypernatremia, in addition to salt gland development, has been a critical feature in the evolution of marine snakes. However, data from intermediate stage (species lacking salt glands but occasionally using salty environments) are lacking to draw a comprehensive picture of the evolution of an euryhaline physiology in these organisms. In this study, we assessed natremia of free-ranging Dice snakes (*Natrix tessellata*, a predominantly fresh water natricine lacking salt glands) from a coastal population in Bulgaria. Our results show that coastal *N. tessellata* can display hypernatremia (up to 195.5 mmol.l^−1^) without any apparent effect on several physiological and behavioural traits (e.g., hematocrit, body condition, foraging). More generally, a review of natremia in species situated along a continuum of habitat use between fresh- and seawater shows that snake species display a concomitant tolerance toward hypernatremia, even in species lacking salt glands. Collectively, these data suggest that a physiological tolerance toward hypernatremia has been critical during the evolution of an euryhaline physiology, and may well have preceded the evolution of salt glands.

## Introduction

Living in seawater entails physiological consequences such as water loss and salt gain, and coping with these constraints represents one of the principal challenges of secondarily marine vertebrates [1]. Accordingly, marine tetrapods (i.e., mammals, birds, turtles, snakes, lizards and crocodiles) display specific adaptations related to the maintenance of osmotic balance. For instance, marine mammals have specialized nephrons which allow highly concentrated urine [2]. Although marine reptiles lack the ability to excrete excess salt in urine, they have evolved salt glands that secrete concentrated salt solution [3], [4].

The widespread relationship between marine life and presence of specific salt-excretory structures, found across very different taxa, strongly suggests that the ability to excrete excess salt has been critical during the invasion of marine environments by tetrapods. However, as with most evolutionary processes, transitional steps are missing and are seldom represented by fossil remains [5]. In addition, crucial characteristics such as physiology and/or behaviour do not print well within the fossil records [5]. Yet, some research works have proposed scenarios of the evolution of an euryhaline physiology during the transition to marine life [6], [7], [8]. For instance, Dunson and Mazzotti [6] have proposed four successive steps that should ultimately lead to an efficient maintenance of the osmotic balance. The first step consists of a primary reliance on behavioural osmoregulation (e.g., frequent obligate fresh water drinking [9]). The second step involves a reduction in salt gain and water loss through permeable surfaces [10], [11], [12]. The third and fourth steps include the evolution of rudimentary salt secreting features and their subsequent development [4], which would ultimately allow exploiting more saline, thus larger, oceanic areas [13].

Clearly, these successive stages would ultimately allow organisms to progressively become emancipated from regular access to fresh water, and thus to thrive in saline environments. Likely, this has led to the conclusion that marine tetrapods could maintain their water balance without consuming fresh water [14], [15]. However, recent investigations have challenged this paradigm. Specifically, the most detailed studies performed on marine snakes have shown that species having a functional salt gland cannot equilibrate their hydromineral balance without access to fresh water [12], [16]. Dehydration in seawater has been shown to occur in amphibious sea snakes (Laticaudine sea kraits) as well as in fully marine species (Hydrophine sea snakes) [12], [16], [17]. In addition, elevated plasmatic sodium concentrations have been measured in various marine snake species [18]–[25]. These studies have led to the hypothesis that the development of a physiological tolerance to hypernatremia may have been an important feature of the evolution of marine snakes [25]. However, data gathered under experimental conditions show that fresh water species lacking salt glands (including coastal presumably salt tolerant species) rapidly accumulate large salt loads when acclimated in brackish and salt water [24], [26]. In most of these cases, the resulting hypernatremia was lethal [24], [26]. Taken together, these elements highlight the lack of information from groups thought to resemble transitional forms between the land and the sea (species lacking salt glands but occasionally using salty environments) to draw a comprehensive picture of the evolution of an euryhaline physiology in these organisms.

Snakes provide a suitable study system with which to clarify the steps that allowed coping with osmotic challenge [13]. Indeed, this lineage displays a unique gradient of habitat use which allows studying groups thought to resemble transitional forms between the land and the sea [13], [27]. Importantly, these transitional steps can be investigated at several phylogenetic scales along a gradient of habitat use [27]. Species that are salt tolerant, but imperfectly marine (i.e., lacking salt glands) represent a powerful opportunity to investigate the early steps of the evolution of an euryhaline physiology. In the current investigation, we examine such a study system. The European Dice snake (*Natrix tessellata*) is a typical semi-aquatic fresh water natricine species that occurs over Eurasia (broadly from Italy to China [28]). Although this species relies primarily on fresh water bodies to forage for fish and amphibians, some populations are known to use, more or less extensively, brackish or saline habitats, thereby offering the possibility to investigate an intermediate step during the evolution to marine life. In this study we report natremia (plasma sodium concentration, an indicator of the osmotic challenge linked to marine life in snakes [25]) measured in free-ranging Dice snakes inhabiting a coastal ecotone between freshwater and the Black Sea in Bulgaria. In combination with a review of plasmatic sodium concentration of snakes, these results are discussed in the light of the secondary transition to marine life in tetrapods.

## Materials and Methods

### Ethics statement

All procedures were approved by French and Bulgarian regulations (Comité d'éthique Poitou-Charentes approval number CE2013-5 to FB; Ministry of Environment and water of Bulgaria permit to YVK: 298/09.03.2011).

### Study species

*Natrix tessellata* is a medium-sized (up to 130 cm [29]) species with an extended Palearctic distribution: from central Europe to northern Egypt and east as far as north-western China [30], [31]. It is a typical semi-aquatic natricine foraging mainly for fish, and to a lesser extent for amphibians in streams, rivers, and lakes [28].

Although the vast majority of *N. tessellata* populations rely on fresh water bodies, few do occur in saline environments along the coasts of the Adriatic Sea [28], [32]–[35], the Ionian and Aegean Seas [36], the Black Sea ([37]–[40], this study) and the Caspian Sea [41], [42]. In most of these cases, *N. tessellata* occurs in brackish waters of lagoons, salt marshes and river mouths.

### Study site

**Figure 1:**
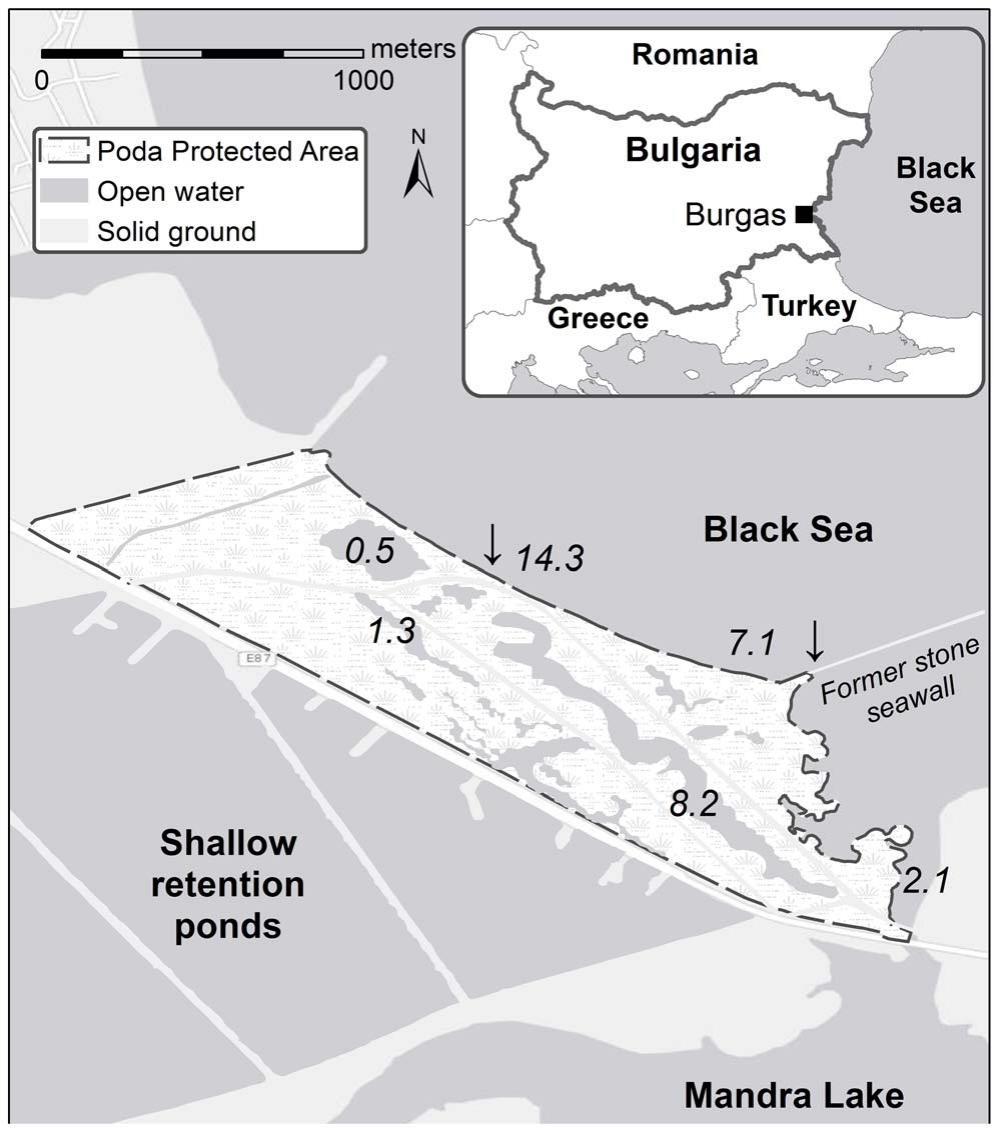
Map of the study area. The upper panel gives the location of the Poda Protected Areas in the vicinity of Bourgas, Bulgaria. Emergent lands are indicated in white, water is indicated in light grey. The lower panel shows the Poda Protected Area (dashed area). Emergent lands are indicated in white and water is indicated in light grey. Numbers designate salinity (‰) recorded for three ponds, two locations on the shore of the Black Sea, and one location at the mouth of the Mandra Lake. The two arrows show sites where tracks from snakes commuting between the land and the Black Sea were observed.

We surveyed a population of Dice snakes on the southern Bulgarian Black Sea coast, in the “Poda” Protected Area (Fig. 1). The Poda wetland (1 km^2^) consists of a coastal ecotone inserted between a large predominantly freshwater reservoir (Mandra Lake) and the Black Sea (Fig. 1). Poda is mainly composed of an alternation of shallow pools of water (usually <1 m deep) intersected by embankment lands and lower, temporarily flooded areas. The proximity of the Mandra Lake and the Black Sea and seasonal climatic fluctuations create a wide variety of aquatic habitats spanning from fresh (<1‰) and brackish (1–10‰) to salt water (>10‰), including some hyperhaline waters during the summer (32–33 ‰, [43], Fig. 1).

### Field procedures

In April 2012, a total number of 19 snakes were captured by hand. Snakes were typically found while basking in the sun. Individuals were measured (snout-vent length [SVL] and total length [TL], ±0.5 cm), weighted (±1 g), and sexed by eversion of the hemipenis. Feeding and reproductive status were assessed by gentle palpation.

Only large adult females (>140 g, N = 13) were blood-sampled to avoid putative detrimental effects of the procedure on smaller individuals, and to avoid sex effects on plasmatic parameters. Blood (∼400 μl) was sampled through cardiocentesis using 30 G-needles. A small fraction (10 μl) of the blood was collected in a micro-capillary tube and centrifuged on site in a minihaematocrit Compur M1101 (Bayer) for 3 min to record haematocrit (packed blood cell volume, %). The remaining blood was centrifuged (3 min at 8,000 G) and the plasma was separated and stored at −25°C until assays were processed. Plasma sodium concentrations were assessed with an ISE module on a Pentra C 200 (Horiba Medical Ltd) compact chemistry analyzer.

At the end of the procedures (usually <30 min), snakes were released at the location of capture.

Water samples were collected from water bodies where *N. tessellata* were observed foraging and/or in the vicinity of which we collected snakes. These sampled stations included 3 ponds within Poda, 2 sites along the coast of the Black Sea as well as 1 site situated at the mouth of the Mandra Lake (Fig. 1). Salinity (‰) was assessed with a Pocket Salt Meter (PAL-ES2, Atago).

### Analyses

We quantified a body condition index (BCI) using residual scores from the linear regression between body size (SVL) and body mass (both variables were log transformed for linearity [44]). We excluded individuals with prey in the stomach from the BCI calculations.

Relationships between natremia and possible correlates (BCI, Hct) were investigated using Spearman rank correlations.

## Results and Discussion

**Figure 2:**
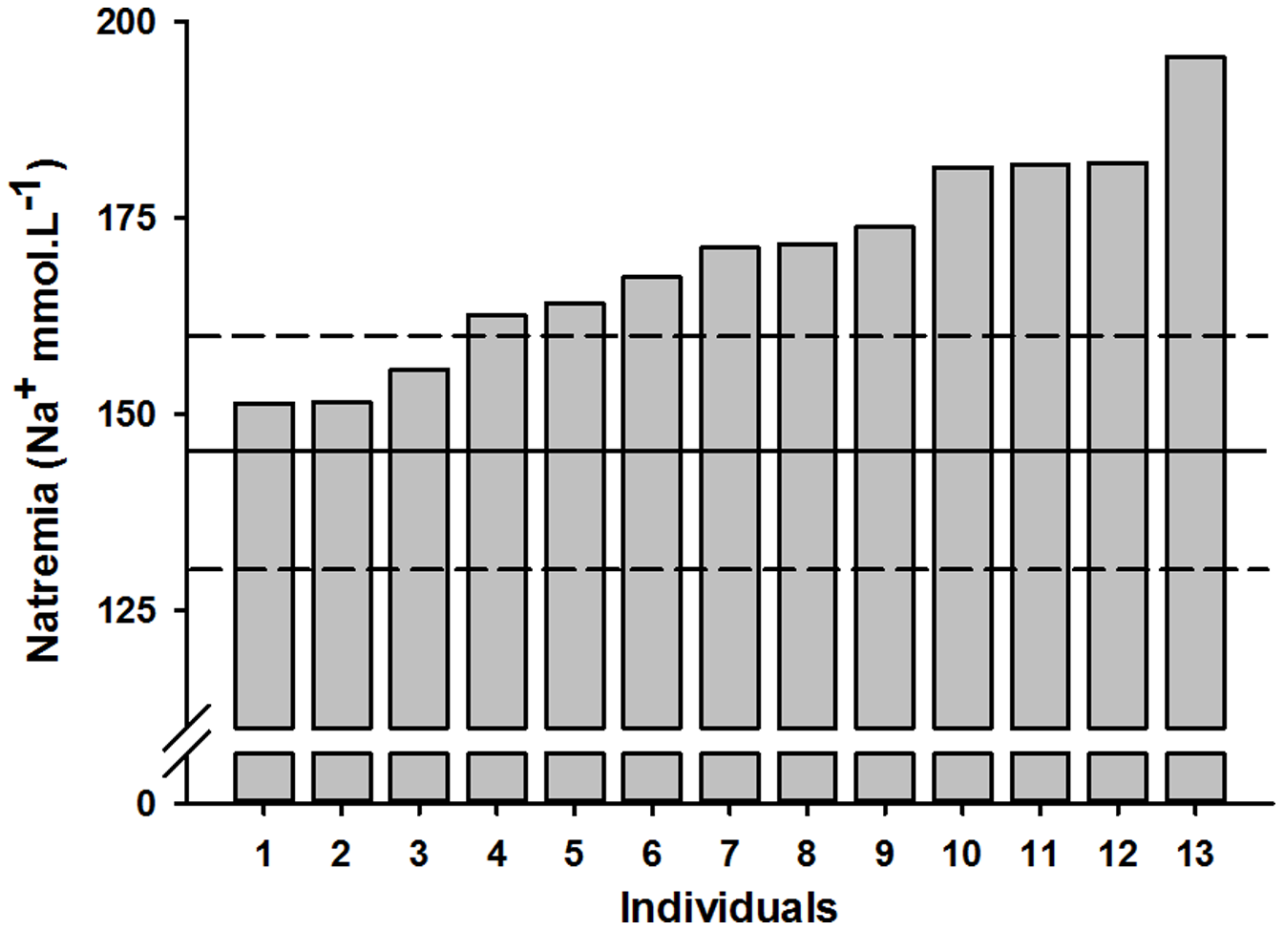
Natremia (plasma sodium concentration) of thirteen free-ranging individual *N. tessellata* captured at Poda Protected Area, Bulgaria. The dashed lines indicate the range of normonatremia (130–160 mmol.l^−1^ [45]) and the horizontal black line indicates mean normonatremia (145 mmol.l^−1^). For clarity, individuals are ranked by ascending order of natremia.

Free-ranging *N. tessellata* display highly variable plasma sodium concentrations (mean 169.9±13.2 mmol.l^−1^) ranging from normonatremia (which range from 130 to 160 mmol.l^−1^ in non-mammalian tetrapods [45]) to hypernatremia (up to 195.5 mmol.l^−1^, Fig. 2). Most individuals (N = 10, 77%) displayed hypernatremia, and only three snakes had values within the range of normonatremia (130–160 mmol.l^−1^ [45]). Classically, deviations of the osmotic balance trigger several behavioural and physiological adjustments in snakes. For instance, dehydrated and/or hypernatremic individuals tend to seclude themselves in well-buffered shelters in order to reduce additional water loss [8], [9]. Such behaviour is usually accompanied by a thermal depression and decreased metabolism which result in a strong reduction in activity levels [46], [47]. Apparently, the high natremia we recorded did not trigger such adjustments in *N. tessellata*. Indeed, all the individuals we captured were basking in the sun, or actively moving in the open, and tried actively to evade capture. Remnants of food were palpated in three individuals that did not display particularly low plasma sodium (155.6, 167.4 and 173.8 mmol.l^−1^ Na^+^ respectively). In addition, BCI was not related to plasma sodium (Spearman rank correlation, *r_s_* = −0.47, *p*>0.05), suggesting an absence of long-term effect of hypernatremia on foraging.

The elevated natremia we found in some individuals could be the result of mere dehydration (i.e., the more dehydrated an individual, the more concentrated its body fluids). However, this hypothesis seems unlikely as we did not find any relationship between plasma sodium and BCI (see above) or haematocrit (Spearman rank correlation, *r_s_* = 0.20, *p*>0.05), two parameters known to correlate with hydration state [17], [48]. Our results rather suggest that free-ranging *N. tessellata* gained salt during their day-to-day activities. Indeed, measurements of environmental salinity in Poda showed that most potential foraging areas were saline (up to 14.3‰, Fig. 1, see also [43]). Likely, Dice snakes foraged in water bodies that were brackish or saline, or indeed at sea (as witnessed by tracks of snakes commuting to the Black Sea, Fig. 1); and gained salt passively through permeable surfaces. Marine snakes display a significant reduction in salt gain and water loss through permeable surfaces [10]–[12], [49], and future studies should assess skin permeability to water and sodium in *N. tessellata* and compare coastal *versus* inland populations.

Voluntary or incidental (e.g., during prey capture) salt water drinking is an additional process that leads to salt gain [15]. Accordingly, marine forms usually display an increased ability to discriminate water salt content and to avoid salt water drinking [12], [16], [26], [50], [51]. We do not know whether *N. tessellata* is able to discriminate water salt content and/or to avoid salt water drinking and such issues need to be clarified. In addition to salt water drinking avoidance, many marine taxa can rely on behavioural osmoregulation such as fresh water drinking. Indeed, dehydrated and hypernatremic marine snakes are known to drink large amounts of fresh water when available to restore osmotic balance [9], [12], [16], [25]. Interestingly, two individuals (151.3 and 162.6 mmol.l^−1^ Na^+^ respectively) regurgitated copious amounts of fresh water upon capture, suggesting that these individuals have drank shortly before. The variety of aquatic habitats found in Poda (fresh, brackish and salt water, Fig 1) over a small spatial scale, may allow hypernatremic Dice snakes to easily access fresh water, and thus to periodically restore osmotic balance. Accordingly, specific environments characterized by low and/or variable salinity may have facilitated evolutionary transitions to marine life in snakes by allowing regular access to relatively fresh water over short time-scales and decreasing the cost of osmotic maintenance [13].

**Figure 3:**
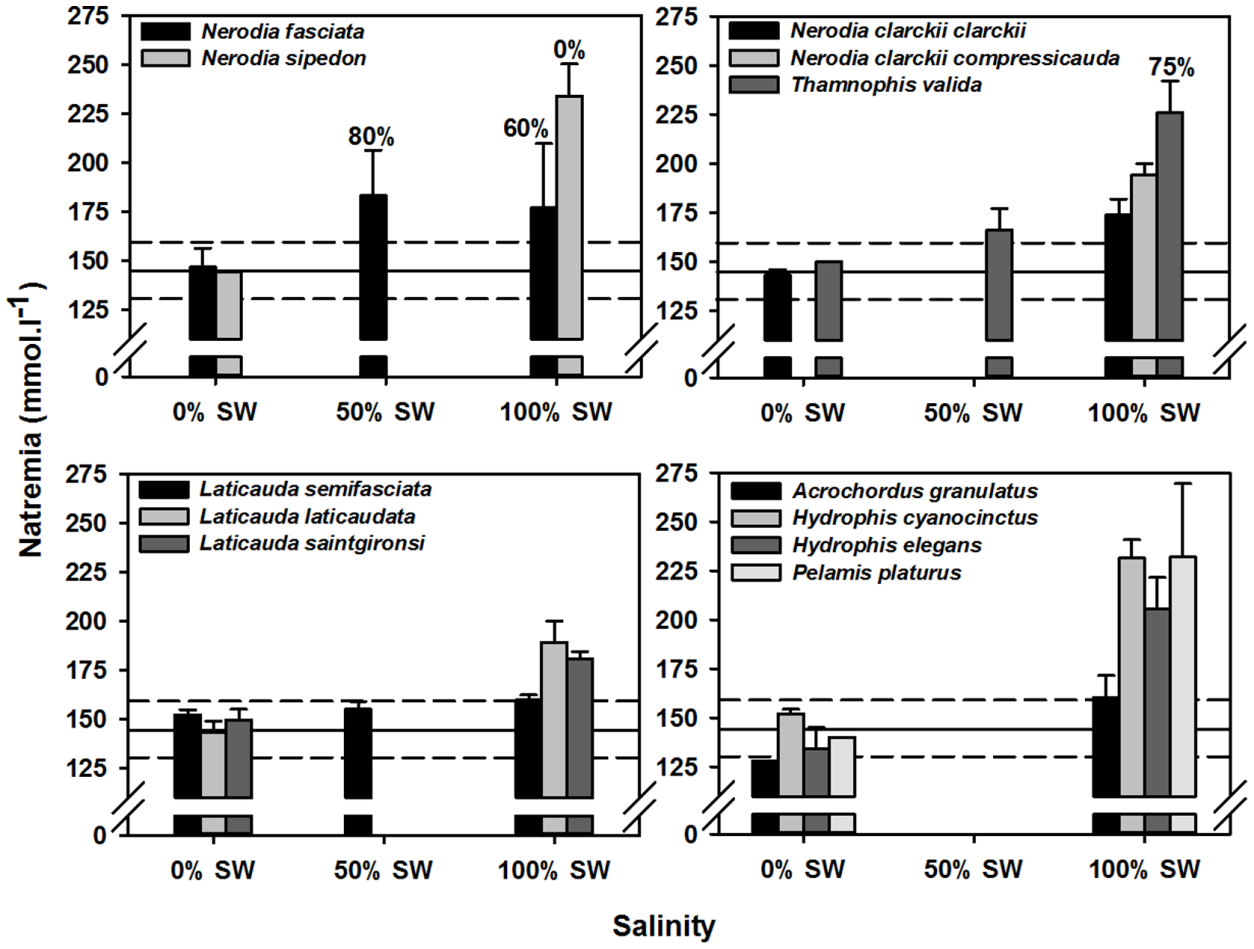
Published data on snake natremia. These data were available from strictly fresh water species (*Nerodia fasciata* and *N. sipedon* [24], [26]), salt tolerant species lacking salt glands (*N. clarckii clarckii*, *N. clarckii compressicauda*, *Thamnophis valida* [24], [26]), amphibious sea kraits with functional salt glands (*Laticauda saintgironsi*, *L. laticaudata*, *L. semifasciata* [24], [25]) and fully marine sea snakes with functional salt glands (*Acrochordus granulatus*, *Hydrophis elegans*, *H. cyanocinctus*, *Pelamis platurus* [18], [19], [21]–[23]. The dashed lines indicate the range of normonatremia (130–160 mmol.l^−1^ [45]) and the horizontal black line indicates mean normonatremia (145 mmol.l^−1^). Numbers above the bars indicate survival rates (no number  = 100%). Data are mean values per species ± SD.

More generally, a review of plasmatic sodium concentration of snakes experimentally maintained in fresh water or seawater gives additional insights to our results (Fig. 3 and references therein). These data suggest that when kept in fresh water, irrespective of their primary habitat (i.e., fresh- *versus* seawater) all species shared similar normonatremia (∼140–150 mmol.l^−1^, Fig. 3). Similarly, in full-strength seawater, plasma sodium increased in all species regardless from their osmoregulatory attributes (i.e., presence/absence of salt glands, Fig. 3, [25]). Importantly, survival differed among species and treatments, with strictly fresh water species (*Nerodia fasciata* and *N. sipedon*) having decreased short-term (i.e., hours) survival in osmotically challenging treatments (Fig. 3). Survival decreased also for salt tolerant species, although to a lesser extent, with only one species (*Thamnophis valida*) out of three having its short-term survival decreased in full-strength seawater (Fig. 3). The other salt tolerant species survived for several days in full-strength seawater. Survival stayed high (100%) in both groups of marine adapted snakes (Fig. 3). Overall, these patterns seem to indicate that along a continuum of habitats use between fresh- and seawater, snake species display a concomitant physiological tolerance toward high plasma sodium, even in species lacking salt glands (Figs. 2 and 3). In turn, this physiological flexibility would allow reducing detrimental effects of salt gain such as decreased activity levels and decreased short-term survival [26]. Such resistance would allow individuals to periodically access fresh water, and hence to occasionally restore their osmotic balance. Ultimately, in marine species having salt glands, such flexibility would allow excreting excess salt when natremia exceeds high thresholds [22], which would substantially decrease energetic costs linked to salt gland functioning [25].

In conclusion, the combination of these data strongly suggest that the development of a physiological tolerance toward deviations of the osmotic balance (e.g., increased plasma sodium) might have been a critical innovation in the evolution of an euryhaline physiology and may well have preceded the evolution of salt glands. Although only few populations of *N. tessellata* are found in saline environments, our results show that these populations may be salt tolerant, and use saline water bodies despite lacking salt glands. In this respect, *N. tessellata* seems a promising study model (i.e., a marine snake prototype) of the secondary transition to marine life in vertebrates.
